# Influenza Vaccine Manufacturing: Effect of Inactivation, Splitting and Site of Manufacturing. Comparison of Influenza Vaccine Production Processes

**DOI:** 10.1371/journal.pone.0150700

**Published:** 2016-03-09

**Authors:** Theone C. Kon, Adrian Onu, Laurentiu Berbecila, Emilia Lupulescu, Alina Ghiorgisor, Gideon F. Kersten, Yi-Qing Cui, Jean-Pierre Amorij, Leo Van der Pol

**Affiliations:** 1 Department of Product Development, Intravacc, Institute for Translational Vaccinology, Bilthoven, The Netherlands; 2 Laboratory of Biotechnology, Cantacuzino National Research Institute, Bucharest, Romania; 3 Unit of Influenza Vaccine Production, Cantacuzino National Research Institute, Bucharest, Romania; 4 Laboratory of Respiratory Viral Infections, Cantacuzino National Research Institute, Bucharest, Romania; 5 Department of Research, Intravacc, Institute for Translational Vaccinology, Bilthoven, The Netherlands; 6 Department of Business Development, Intravacc, Institute for Translational Vaccinology, Bilthoven, The Netherlands; Icahn School of Medicine at Mount Sinai, UNITED STATES

## Abstract

The aim of this study was to evaluate the impact of different inactivation and splitting procedures on influenza vaccine product composition, stability and recovery to support transfer of process technology. Four split and two whole inactivated virus (WIV) influenza vaccine bulks were produced and compared with respect to release criteria, stability of the bulk and haemagglutinin recovery. One clarified harvest of influenza H3N2 A/Uruguay virus prepared on 25.000 fertilized eggs was divided equally over six downstream processes. The main unit operation for purification was sucrose gradient zonal ultracentrifugation. The inactivation of the virus was performed with either formaldehyde in phosphate buffer or with beta-propiolactone in citrate buffer. For splitting of the viral products in presence of Tween^®^, either Triton^™^ X-100 or di-ethyl-ether was used. Removal of ether was established by centrifugation and evaporation, whereas removal of Triton-X100 was performed by hydrophobic interaction chromatography. All products were sterile filtered and subjected to a 5 months real time stability study. In all processes, major product losses were measured after sterile filtration; with larger losses for split virus than for WIV. The beta-propiolactone inactivation on average resulted in higher recoveries compared to processes using formaldehyde inactivation. Especially ether split formaldehyde product showed low recovery and least stability over a period of five months.

## Introduction

Yearly, genetic shift and drift of influenza virus [1] necessitate the manufacturing of high numbers of influenza vaccine with yearly adapted vaccine strains. Although in potential, the worldwide vaccine production capacity of 850 million doses per year [1–3] is nearly matching the seasonal demand for influenza vaccine, this amount is not sufficient to cover demands for a pandemic outbreak. The current influenza vaccines on the market are live attenuated influenza vaccines and inactivated influenza virus vaccines [4,5]. Inactivated influenza virus vaccines include whole inactivated virus vaccines (WIV), split virus vaccines, subunit vaccines (split virus from which the nucleocapsid is removed) and virosomal influenza vaccines (reconstituted virus envelope material) [4,5]. Beside vaccines made from the influenza virus produced in eggs or mammalian cells, a subunit vaccine based on recombinant haemagglutinin (HA) produced in insect cells is licensed. Each of these vaccines has its specific advantages and disadvantages as reported elsewhere: [5]

The classical WIV production starts with influenza virus growth in eggs followed by a clarification step and zonal ultracentrifugation. Subsequently, the intermediate bulk is inactivated and formulated before sterile filtration and fill & finish. In the case of split vaccine the virus is split and the splitting agent removed prior to formulation and sterile filtration. Influenza subunit vaccines contain additional purification steps to remove the nucleocapsids and lipids before formulation.

At the expense of immunogenicity [6–9] split influenza vaccines, and also subunit influenza vaccines, are more common nowadays than WIV vaccines, because subunit vaccines are less associated with side effects [5,10,11].

Initial splitting technology, introduced in the 1960s, was based on diethyl-ether extraction of the virus [12–14]. However, the use of volatile diethyl-ether (ether) has several drawbacks, such as risk of explosion, local toxicity (irritation of skin and eyes) as well as toxicity after repeated or prolonged exposure resulting in organ damage [15,16]. Moreover, the use of ether resulted in difficulties with the quantification of HA in the split product [16]. As a result, currently, most of the split influenza vaccines are produced by alternative methods including splitting by de-oxy-cholate (Afluria, Flulaval^®^, Fluarix^®^) and Triton^®^X-100 (Fluzone^®^).

**Fig 1:**
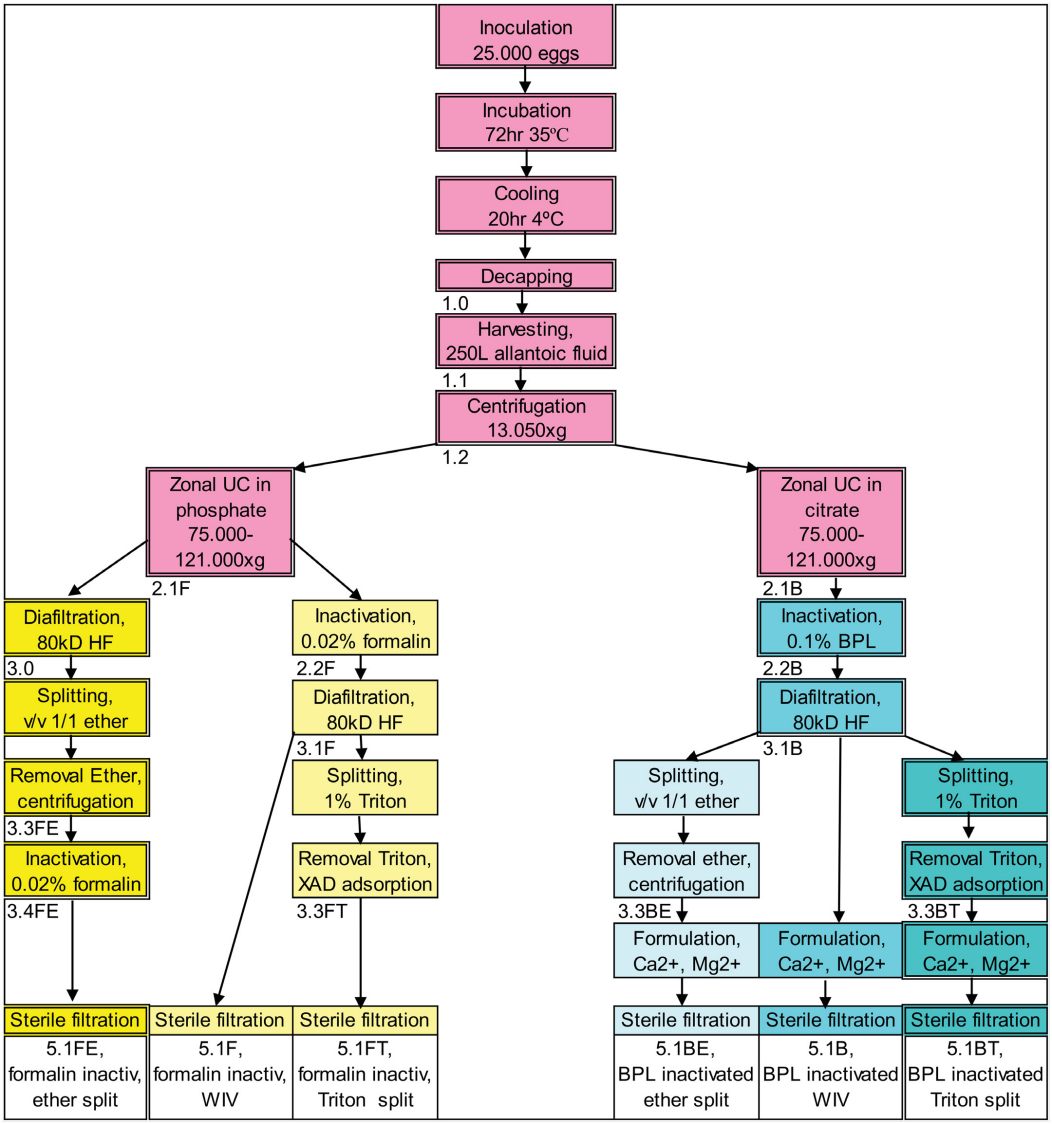
Overview of the process flows, starting with inoculation of 25.000 eggs and resulting in 6 vaccine products. In the boxes the unit operations are presented. Fraction identification number is written below the unit operation box. Fraction 1.2 (clarified allantoic fluid) was equally divided over the six process streams. The processes from left to right, with the end product, given in the bottom boxes below the unit operation ‘Sterile Filtration’: 5.1FE standard Cantacuzino Institute process for H3N2 strain, 5.1F Whole Inactivated Virus (WIV) inactivated by formaldehyde, 5.1FT formaldehyde inactivated, Triton split virus product, 5.1BE beta-propiolactone (BPL) inactivated, ether split virus product, 5.1B WIV inactivated by BPL, 5.1BT standard Intravacc process.

The aim of the current study is to evaluate the impact of the inactivation and splitting procedure on product composition and recovery, at production scale, to support transfer of influenza vaccine production technology. Intermediate product as well as final products were characterized and compared among the different processes studied. At the Cantacuzino Institute (Cantacuzino) the manufacturing processes based on Intravacc protocols (comprising beta-propiolactone inactivation and splitting by Triton) was performed head-to-head to their standard manufacturing process (formaldehyde used to inactivate and ether to split). This resulted in six processes: two commonly performed split processes, two hybrid split processes and two WIV processes, as shown in the overview in Fig 1.

## Materials & Methods

### Production of influenza WIV and split vaccines

Six different influenza vaccine batches of bulk vaccine product were produced starting from one batch of clarified allantoic fluid. The main characteristics of the performed production processes are summarized in Table 1, whereas the respective accompanying process flowcharts are presented in Fig 1.

**Table 1:** Overview of the vaccine bulk products produced including the differences in the applied unit operations.

Investigated/Used Unit Operations	Products					
	Split 5.1FE	WIV 5.1F	Split 5.1FT	Split 5.1BE	WIV 5.1B	Split 5.1BT
**Inactivation formalin**	√	√	√			
**Inactivation BPL**				√	√	√
**Splitting ether**	√			√		
**Splitting Triton**			√			√
**Formulation PBS**	√	√	√			
**Formulation PBS+Mg+Ca**				√	√	√

The process in column ‘Split 5.1FE’ is Cantacuzino standard and the process ‘Split 5.1BT’ is Intravacc standard.

**Note**: In the case of H3N2 reassortant, used for this study, it was experienced that inactivation by formaldehyde, followed by treatment with ether, did not result in split product to the desired split extend (60% to 80% as specified for the product of Cantacuzino). Performing the inactivation with formaldehyde, after splitting the virus with ether (Fig 1), yields a suitable influenza split vaccine product.

Good Manufacturing Practices (GMP) compliant facilities of Cantacuzino were used to produce the influenza vaccine batches. The upstream manufacturing process of influenza vaccines consists of the following unit operations: inoculation of 11 days old embryonated eggs (local supplier Romania) with influenza seed virus (Influenza A/H3N2 of strain A/Uruguay/716/2007 X-175C, Solvay Weesp, The Netherlands), incubation of inoculated eggs for 72 hr at 35°C and overnight cooling to 2–7°C. The allantoic fluid was harvested and clarified by centrifugation. The clarified harvest (Fig 1, fraction 1.2) was used as starting material and divided in equal amounts over the six different purification processes.

For the fraction to be inactivated by formaldehyde zonal ultracentrifugation (ZUC) was performed in 60% sucrose in phosphate buffered saline (PBS) (Invitrogen) (Fig 1, fraction 2.1F). The ZUC of the fraction to be inactivated by beta-propiolactone (BPL) was performed in 60% sucrose in 125 mM sodium citrate buffer pH 7.8 (Invitrogen) (Fig 1, fraction 2.1B), since BPL induced inactivation requires a higher buffer capacity to prevent a major pH reduction [17,18].

Formaldehyde inactivation (24 hr at 2–7°C) was performed at a final concentration of 0.02% formalin, whereas BPL based inactivation (24 hr, 18–22°C) was with a final BPL concentration of 0.1%.

Sucrose was removed to less than 3% (w/w) by 10 times diafiltration against PBS, using 80 kD MWCO hollow fiber filters (Microza Membranes, Pall).

For the ether-tween split products, first Tween^®^ 80 (polysorbate 80, Merck KGaA) was added to the bulk to a concentration of 1.25 mg/mL and then combined with an equal volume of ether (Diethyl Ether, Merck KGaA) while stirring at 4°C. The two phases were separated by centrifugation (CS 50 Centrifugal extractor, CINC, Germany) and ether (in the top phase) was removed by pumping, while the removal of ether was completed by subsequent evaporation (Fig 1, fractions 3.3FE and 3.3BE).

Fractions 3.1F and 3.1B were split by 1% Triton^™^ X-100 (Sigma-Aldrich) in presence of 500 mg/L Tween during stirring for 1 hr at 20°C. Detergents were removed from the fractions (Fig 1, fraction 3.3FT and 3.3BT) by recirculation over a column (XK50/20 column, GE healthcare) filled with Amberlite^™^ XAD-4 (Sigma-Aldrich), at 20–25°C and at linear flow of 0.5 cm/min during 3–6 hours. Removal was monitored by UV280 until UV-absorption did not decrease further. The final products inactivated by formaldehyde were all in PBS; the final products inactivated by BPL were all in PBS, to which 1% subunit buffer B (Invitrogen) was added before sterile filtration resulting in a final concentration of 0.5mM Mg^2+^ and 0.9 mM Ca^2+^ to stabilize neuraminidase (NA).

### Analysis methods for main characteristics

The release tests for influenza vaccine for human use, as specified by the World Health Organization (WHO) and European Pharmacopeia (EP) were performed on the bulk products and on several intermediate product fractions. Table 2 is listing the assays including the requirements for the vaccine bulk product. More detail on the test methods is available in S1 File.

**Table 2:** Parameters of the bulk material quantified/determined, including specification and reference to method used.

Parameter/quality attribute	Specification [unit]	Method [reference]
Haemagglutinin antigen concentration	≥ 90 [μg/mL]	Single Radial ImmunoDiffusion (SRID) assay [EP 2.7.1, Immunochemical methods (2004)],[19]
Haemagglutinin antigen	Present conform strain	SRID assay [EP 2.7.1, Immunochemical methods (2004)], [19]
Neuraminidase antigen presence and activity	Present conform strain	Neuraminidase inhibition assay [EP 01/2008:0159]
Total Protein	< 600 [μg /100 μg HA]	Petersen colorimetric assay [20]
Ovalbumin	< 2 [μg /100 μg HA]	Enzyme Linked Immuno Sorbent Assay (ELISA) [EP 2.7.1, Immunochemical methods (2004)]
Endotoxins	≤ 200 [IU/mL]	Limulus Amoebocyte Lysate test [EP 2.6.14, Bacterial endotoxins]
Residual Infective Virus	Inactive	Immunological [EP 0159]
Sterility	Sterile	Membrane filtration [EP 2.6.1]
pH	6.9–7.7	[EP 2.2.3, Potentiometric determination of pH]
Beta-Propiolactone	< 10 [ppm]	Nuclear Magnetic Resonance (NMR)
Free formaldehyde	≤ 0.2 [g/L]	[EP 2.4.18, Free formaldehyde]
Triton X-100	≤ 100 [μg/100 μg HA]	1H-NMR spectroscopy [EP 2.2.34, Thermal Analysis]
Hydrodynamic size	Not applicable	Radius by Dynamic Light Scattering
Sub microscopic morphology	Not applicable	Electron microscopy

### Analysis methods for additional characterization of (intermediate) products

#### Dynamic Light Scattering

Dynamic Light Scattering (DLS) (Malvern Zetasizer, Ver. 6.20, Malvern Instruments Ltd) was used to determine the influenza antigen particle hydrodynamic radius in the different batches. Particle size distribution by intensity (more relevant for bigger particles) and by mass (more relevant for smaller particles) were evaluated, as well as the polydispersity index (PDI) which is a measure for size distribution; a value below 0.05 is representative for a monodisperse sample, whereas values above 0.7 indicate a broad size distribution [21]

#### SDS-PAGE and Mass Spectrometry

The heterogeneously and heavily glycosylated HA-protein is resulting in diffuse bands during Sodium Dodecyl Sulfate—Poly Acrylamide Gel Electrophoresis (SDS-PAGE), which complicates the interpretation. Facilitating the evaluation of the product fractions and the quantification of HA-protein, the sample preparation for SDS-PAGE was performed with and without de-glycosylation, according to the alternative HA quantification (AHQ) method of Harvey [22].

The identity of the each major band on the gel was confirmed by mass spectrometry (MS) as described by Meiring and colleagues [23]; the acquired data were qualified using the protein database UniprotKB/SwissProt (available from http://www.uniprot.org).

The relative density of bands containing HA protein (HA1+HA2) on the SDS-PAGE gel after de-glycosylation was quantified against the total protein loaded on the gel.

### Stability evaluation

The stability of HA in the vaccine bulks during storage at 2–8°C over a period of 5 months was evaluated by measurement of the HA content by SRID [19]. The HA preservation is expressed as percentage of the HA concentration in the bulk immediately after production.

## Results and Specific Discussions

### Main characteristics of the six bulk products

Based on standard tests the six vaccine bulk products were analyzed. The concentration of total protein and ovalbumin in the starting material, the clarified harvest before ZUC (fraction 1.2) is presented in Table 3. The haemagglutinin (HA) concentration was not measured (below detection limit of the test), The results for HA, total protein and ovalbumin in the fraction after ZUC in phosphate buffer and after ZUC in citrate buffer are given in Table 3 as well. For total protein and HA results are similar; ovalbumin concentration in fraction 2.1B (after ZUC in citrate) is lower than in fraction 2.1 F (after ZUC in phosphate). In the 5.1 bulk fractions the average ovalbumin concentration is rather similar with 3.1 μg/mL (stdev 0.5 μg/mL), as presented in Table 4, together with the other main characteristics of the bulks.

**Table 3:** The main characteristics of the starting material before (fraction 1.2) and after ZUC, i.e, fraction 2.1F and 2.1B. Haemagglutin (HA) and total protein concentration are similar; ovalbumin concentration after ZUC in citrate buffer is lower than after ZUC in phosphate buffer.

		Clarified harvest, before ZUC	ZUC in phosphate buffer	ZUC in citrate buffer
**Parameter**	**[unit]**	**1.2**	**2.1F**	**2.1B**
**Haemagglutinin (HA)**	[μg/mL]	No data	820	818
**Total protein**	[μg/mL]	2134	2809	2861
**Ovalbumin**	[μg/mL]	947	12	5.2

**Table 4:** The main characteristics of the products from the six different downstream processes. If applicable the requirements are listed. All products comply with the requirements, except product 5.1FE that has low antigenic HA content

row			Ether split formaldehyde inactivated virus	Whole formaldehyde inactivated virus	Formaldehyde Triton split virus	BPL inactivated ether split virus	Whole BPL inactivated virus	BPL inactivated Triton split virus
1	**Parameter, quality attribute**	**Requirement,[unit]**	**5.1FE**	**5.1F**	**5.1FT**	**5.1BE**	**5.1B**	**5.1BT**
2	**Haemagglutinin (HA)**	> 90,[μg/mL]	**83**	165	192	331	218	**333**
3	**Total protein**	[μg/mL]	625	645	**397**	**840**	735	755
4	**HA-protein by AHQ**	[μg/mL]	264	**248**	271	**388**	317	362
5	**Total protein/HA**	≤ 600,[μg/100 μg HA]	**749**	390	**206**	254	338	227
6	**Neuraminidase (NA)**	Present [μg/mL]	4.9	**4.0**	4.1	**6.8**	5.5	6.2
7	**Ovalbumin**	[μg/mL]	2.8	2.7	3.7	**3.8**	**2.6**	3.1
8	**Ovalbumin/HA**	≤ 2 [μg/100 μg HA]	**3.3**	1.6	1.9	1.2	1.2	**0.9**
9	**HA/total protein**	[μg/100 μg TP]	**13**	26	**48**	39	30	44
10	**NA/total protein**	[μg/100 μg TP]	0.8	**0.6**	**1.0**	0.8	0.7	0.8
11	**Ovalbumin/total protein**	[μg/100 μg TP]	0.4	0.4	**0.9**	0.5	0.4	0.4
12	**Sucrose/total protein**	[mg/100μg TP]	4.4	4.3	**6.3**	2.1	3.7	**1.3**
13	**Sucrose**	[mg/mL]	28	28	25	18	28	**10**
14	**Z.ave radius**	[nm]	64	**82**	54	**52**	78	66
15	**Polydisp.index Z.ave**		0.18	0.11	**0.28**	0.18	**0.04**	0.23

In the upper row a short description of the product is given; the code as used in Fig 1 is stated in row numbered 1. Bold numbers present the highest and lowest test value of the products.

#### Haemagglutinin

Since a human dose has to contain 15 μg HA/strain per 0.5 mL injection volume the bulk requirement for HA concentration, as measured by SRID, is ≥ 90 μg/mL. As shown in Table 4, almost all products meet the target specification for HA content. The formaldehyde inactivated, ether split product (5.1FE), is the only bulk not meeting this requirement. The 5.1FE bulk has a significant lower HA concentration (83 μg/mL) than the other products (165–333 μg/mL) (Table 4, row 2). In addition, because the total protein concentration of the 5.1FE product is in the same range as the protein concentration of the other products (Table 4, row 3), the HA/protein ratio of product 5.1FE is lower than the HA/protein ratio of the other five products (Table 4, row 9). Finding only 13 μg HA/100 μg for product 5.1FE (Table 4, row 9), suggests that the purity has dramatically decreased compared to the ZUC phase. After ZUC (Table 3) the total protein concentration was 2835 +/- 37 μg/mL (Fig 1, average of 1.2F and 1.2B) of which HA concentration 819 +/-1 μg/mL (Fig 1, average of 1.2F and 1.2B), i.e. 28 μg HA/100 μg total protein.

In contrast to the ether split and subsequently formalin inactivated 5.1FE bulk product, the first BPL inactivated and then ether split bulk, 5.1BE, does not show such a low HA/protein ratio (Table 4, row 9).

Comparably, in their investigations on influenza split vaccine products prepared by ether splitting and formaldehyde inactivation, Johannsen e.a. [24] found that the SRID underestimated the HA content by 25–50%, which was attributed to the aggregated state of the product, since an additional treatment to dissolve the aggregates (eg. octyl glucoside +Tween-ether or sonification in 1% Mulgofen in 0.9 M phosphate buffer pH 7.2) increased the HA content measured by SRID. In our here described study, the DLS results did not indicate significant more aggregation in the 5.1FE product compared to the other batches. The apparent low recovery of the formaldehyde ether split product may be related to other specific chemical or physical changes of the HA protein.

#### Neuraminidase

The European Medicines Agency (EMA) specification for influenza vaccines requires the presence of NA as qualitative specification. From Table 4, row 6, it can be deduced that all produced bulks comply with this requirement; the NA concentrations in the final fractions (Fig 1, 5.1 fractions) ranged from 4.0 to 6.8 μg/mL.

#### Ovalbumin

As shown in Table 4, row 7, the ovalbumin concentration in the produced bulks was in the range of 2.6 to 3.8 μg/mL. The only bulk that deviated from the specification of less than 2 μg ovalbumin per 100 μg antigenic HA (Table 3, row 8), was the ether split formaldehyde inactivated product (5.1FE) that contained 3.3 μg ovalbumin per 100 μg HA (Table 3, row 8). However: after purification by ZUC (Fig 1, fractions 2.1F and 2.1B), per 100 μg HA only circa 1 μg of ovalbumin was present (Table 4), indicating that antigenic HA is lost in the unit operations succeeding ZUC.

#### Size

The DLS measurements (Table 4, row 15) of the bulk products showed that whole inactivated virus products contain larger particles than the split products. Whereas WIV vaccines 5.1F and 5.1B showed a mean radius of 78–82 nm, the split products radius ranged from 52 to 66 nm. Of the manufactured products, the BPL WIV (5.1B) was most uniform in structure with a monodispersity (PDI) of 0.04 versus PDI values in the range of 0.11 to 0.28 for the other bulks.

### Recoveries of the six downstream processes

In the intermediate product fractions collected from the process steps before ZUC, no reliable HA concentration could be measured since the concentrations were below the detection limit of the SRID test. The recoveries based on HA quantity are therefore calculated relative to the amount present (Table 3) in the fractions after ZUC (Fig 1, fractions 2.1F and 2.1B).

**Fig 2:**
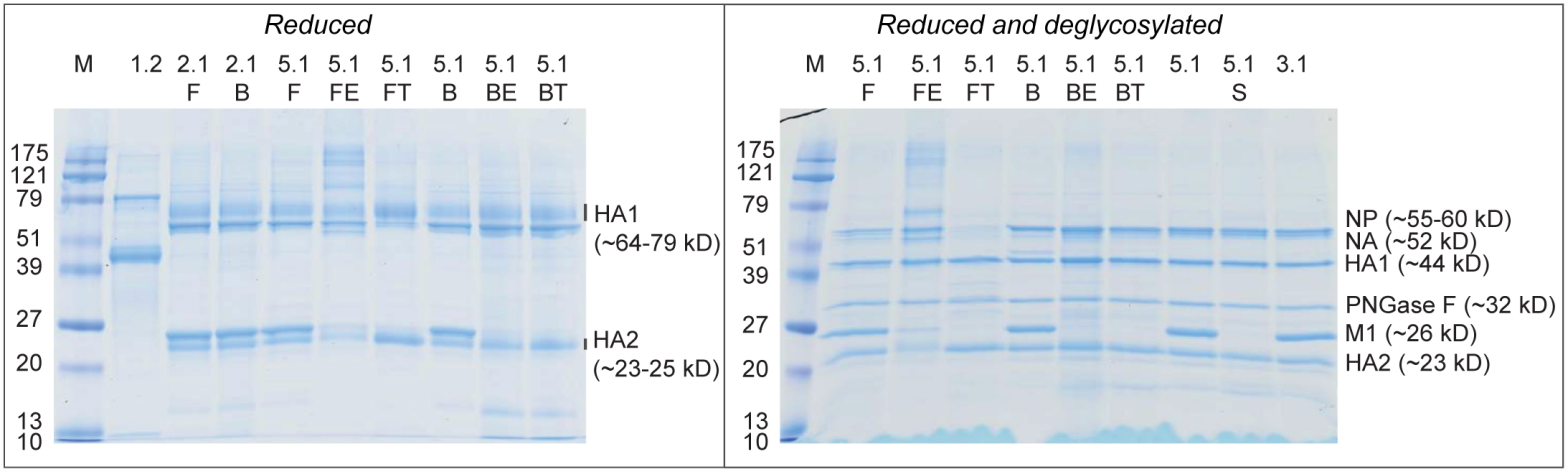
SDS PAGE of reduced and reduced plus de-glycosylated samples as a fingerprint of principle proteins present. Lanes M were loaded with marker proteins, with the corresponding molecular weight presented to the left. The fraction sample identity (Fig 1) is noted above the lane. Left gel: 1.2 before ZUC, 2.1F after ZUC in phosphate, 2.1B after ZUC in citrate, followed by the six bulks (5.1F, 5.1FE, 5.1FT, 5.1B, 5.1BE and 5.1BT); the migration distance of heavily glycosylated HA proteins varies, causing diffuse bands. In such a case the HA1 band range (~64–79 kD) may be difficult to discriminate from the Nucleoprotein band (~55–66 kD) and the HA2 band range (~23–25 kD) may cover the location of M1 band (~26 kD) as reported by Harvey [22]. After de-glycosylation the HA bands are more distinct and migration distance has increased (right gel, bulks 5.1F, 5.1FE, 5.1FT, 5.1B, 5.1BE and 5.1BT). NP and M1 protein bands have not changed position due to the applied de-glycosylation. In the lanes to the right of the right gel, for comparison products prepared at Intravacc site were applied: 5.1 is WIV BPL inactivated bulk, 5.1S is BPL inactivated Triton split bulk and 3.1 is BPL inactivated influenza before splitting with Triton

ZUC is actually the most effective purification step of the process as indicated by the SDS-PAGE results presented in Fig 2 (lane 1.2 before ZUC and lanes 2.1F and 2.1B after ZUC). As a consequence the ratio total protein to HA-protein concentration does not change much after this unit operation, in other words the recovery of total protein is indicative for the recovery of HA-protein.

The recoveries per unit operation based on antigenic HA, and based on total protein are presented in Table 5.

**Table 5:** The recoveries after each unit operation for the six downstream processes, based on total protein (tot.protein) and HA quantities, relative to the fraction after zonal ultracentrifugation (Fig 1, fraction 2.1F and 2.1B). Noteworthy is the difference in HA recovery versus total protein recovery after sterile filtration of the ether split formaldehyde inactivated product FE: 32% versus 72% in product 5.1 after SF, which cannot be attributed to the test variation of 7.5% for total protein and 20% for SRID test.

**Formaldehyde inactivated products**		**ether split virus**		**whole virus**		**Triton split virus**	
		FE	FE	F	F	FT	FT
		tot.protein	HA	tot.protein	HA	tot.protein	HA
description	fraction	%	%	%	%	%	%
after zonal	2.1	100%	100%	100%	100%	100%	100%
after inactivation	2.2	Na	na	115%	95%	115%	95%
after DF	3.0, 3.1	87%	86%	93%	104%	93%	104%
after split	3.3	94%	96%	na	na	70%	85%
after SF	5.1	72%	32%	52%	49%	49%	72%
Total of all unit operations		58%	**27%**	56%	**49%**	36%	**60%**
**Beta-PropioLactone inactivated products**		**ether split virus**		**whole virus**		**Triton split virus**	
		BE	BE	B	B	BT	BT
		tot.protein	HA	tot.protein	HA	tot.protein	HA
description	fraction	%	%	%	%	%	%
after zonal	2.1	100%	100%	100%	100%	100%	100%
after inactivation	2.2	103%	103%	103%	103%	103%	103%
after DF	3.0, 3.1	100%	102%	100%	102%	100%	102%
after split	3.3	68%	85%	na	na	77%	91%
after SF	5.1	84%	90%	69%	71%	52%	67%
Total of all unit operations		58%	**81%**	71%	**74%**	41%	**64%**

The upper part of the table presents all processes using formaldehyde inactivation, the lower part presents all processes including BPL inactivation. The fraction numbers relate to the phase after a unit operation (Fig 1).

na: not applicable (Fig 1). “Total of all unit operations” is the final recovery result after all unit operations of the downstream process starting from the zonal centrifugation fraction at 100%

DF: diafiltration

SF: sterile filtration

Since the unit operations after zonal centrifugation (2.1) are polishing steps, the purity (ratio antigenic HA to total protein) remains constant if HA loss and protein loss are in the same order of magnitude. This is indeed the case except for ether split virus, were a relative large loss of antigenicity was measured after sterile filtration (fraction 5.1FE). The opposite was observed for Triton split virus: HA purity increased after sterile filtration (fraction 5.1FT). This difference in protein composition is confirmed with gel electrophoresis (Fig 2), showing that 5.1FE contains other proteins than HA, whereas fraction 5.1FT contains mainly HA.

The most pronounced differences in the recoveries occur after splitting and after sterile filtration (SF). In order to identify discrepancies we have used a conservative procedure applied to the log-ratios of the proportional decrease in HA to the proportional decrease in protein [S2 File].

This procedure indicates that the discrepancy found for product 5.1 FE is too large to be attributed to chance. The method used controls the false positive rate (FDR) by means of the Benjamini-Hochberg procedure [25] in order to prevent the detection of spurious discrepancies. On the other hand, because the method is somewhat conservative, some less obvious discrepancies may have escaped us using this method. For example the BPL inactivated products seem more similar in HA and total protein recovery over SF than the formaldehyde inactivated products.

On average the HA recovery of the three BPL inactivated products is higher than for the three formaldehyde inactivated vaccine products.

### Haemagglutinin content based on SDS-PAGE alternative HA-protein quantification method

In Fig 2 the results of SDS-PAGE under reducing conditions without de-glycosylation (left gel) and with de-glycosylation (right gel) of the protein samples are presented. As expected the de-glycosylated HA protein bands are more distinct and at increased migration distance (right gel) compared to glycosylated HA bands in the left gel.

The AHQ method revealed apparent higher HA-protein concentration estimation for all samples (Table 4, row 4). However, for most bulk products the differences between the AHQ based HA estimations and SRID results were within the variation of the tests (7.5% for Lowry based Petersen total protein and 20% for SRID assay). The exception is the 5.1FE (ether split, formaldehyde inactivated) bulk product that showed an AHQ based HA-protein estimation of approximately three times higher than the HA concentration measured by SRID.

Applying the above mentioned AHQ method for HA-protein, 5.1FE product would meet requirements related to the HA-protein concentration, but the antigenicity of the HA-protein present is unclear. The ratio antigenic HA to HA-protein of the bulks varies from 70–90%, but 5.1FE specific HA antigenicity is only 31%.

### Characterization and comparison of (intermediate) product fractions by SDS-PAGE and MS

SDS-PAGE (Fig 2, left gel) of clarified harvest (Fig 1, fraction 1.2) and the fractions after ZUC in phosphate buffer (Fig 1, fraction 2.1F) and in citrate buffer (Fig 1, fraction 2.1B) confirms the effective purification by ZUC: the abundant protein bands (MW ovalbumin circa 43 kD) in the lane of 1.2 sample are not visible in lanes of 2.1F and 2.1B samples, and the clear bands in the lanes of 2.1F and 2.1B are not recognized in the lane of 1.2 sample of clarified harvest. Lanes with fraction 2.1F and 2.1B (virus after ZUC in respectively phosphate buffer and citrate buffer) seem identical in protein composition; no influence of the buffer is noticed. Fractions 3.0 (whole virus), 3.1F (formaldehyde inactivated whole virus) and 3.1B (BPL inactivated whole virus) evaluated by SDS-PAGE (S1 Fig), resemble the WIV product fractions 5.1F and 5.1B; no major change in protein composition due to inactivation or sterile filtration is visible.

SDS-PAGE analysis of the de-glycosylated samples (Fig 2) clearly shows differences in protein band pattern between the ether split (5.1FE) and Triton split (5.1FT) formaldehyde inactivated products. The 5.1 FE ether split product band pattern displays relative large amounts of protein in the higher molecular weight zone and shows some undissolved material in the sample application well. To a lesser extent bands in MW range of 100–220 kD are also present in the sample of the BPL inactivated ether split product 5.1BE. The lanes with the whole inactivated virus products 5.1F and 5.1B display no such large entities. With the H3N2 vaccine production process performed at Intravacc similar results were obtained, as can be seen from the products applied in lanes 5.1 (BPL inactivated WIV bulk), 5.1S (BPL inactivated, Triton split bulk) and 3.1 (BPL inactivated WIV before sterile filtration) in Fig 2, gel to the right.

The nucleoprotein (NP) band (55–60 kD) shows a comparable density to the HA1 band (~44 kD) for all products (Fig 2), except for product 5.1FT, that has no visible NP band.

**Fig 3:**
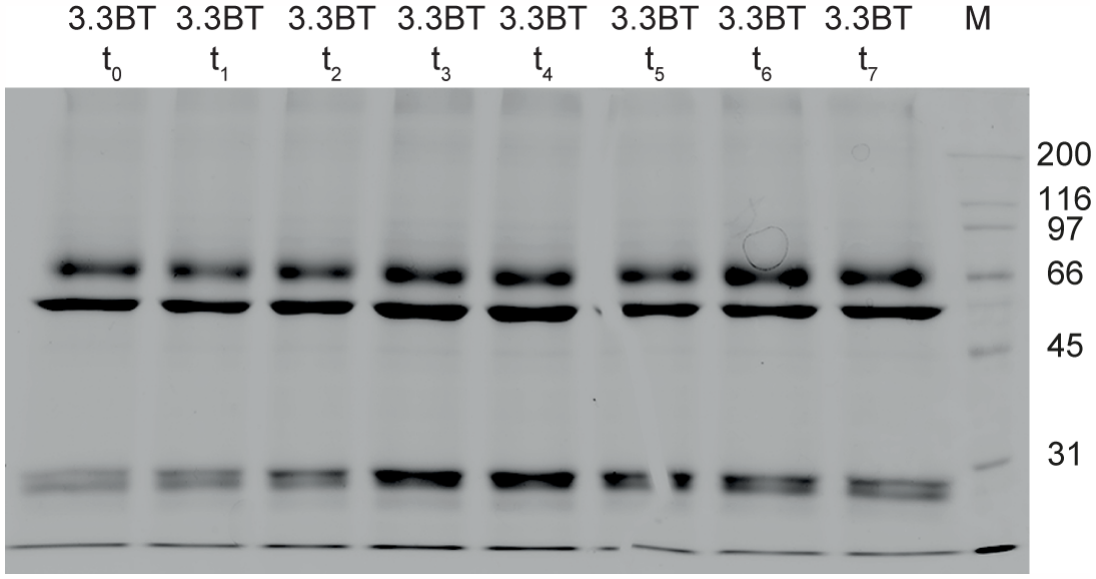
SDS PAGE of samples (reduced) taken during removal of Triton of BPL inactivated virus (3.3BT). Samples were taken approximately every half hour (start at t = 0, last sample at t = 7). Lane 3.3BT t_0_ fraction before removal of Triton, lane 3.3BT t_7_ fraction after removal of Triton. Lane M presents molecular weight (MW) markers, with right of lane M the MW indicated in kD. M1 matrix protein (~26 kD) band is present in both lanes, as are all other clearly visible bands, indicating that no major protein is lost during removal of Triton.

**Fig 4:**
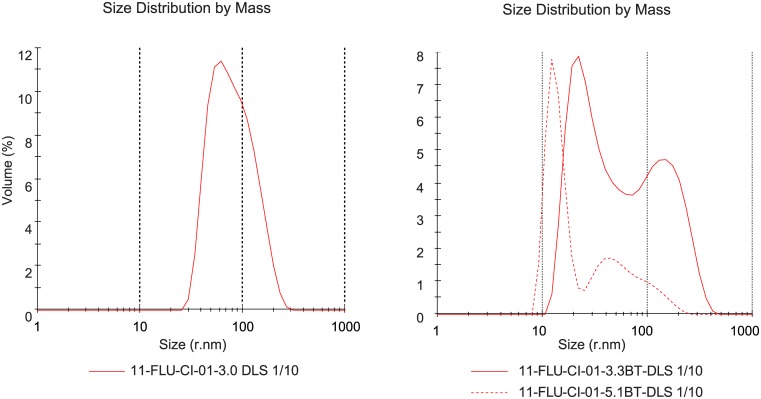
DLS results of fraction before split and after split, before and after sterile filtration. Left panel presents purified live influenza virus fraction 3.0 (Fig 1). Right panel, red solid curve, presents results of fraction 3.3BT, BPL inactivated virus, after splitting and removal of Triton. Clearly two populations are present indicating the splitting of the virus was effective. After sterile filtration of this fraction (5.1BT, red dotted curve), significantly less volume % of large entities is present.

Matrix protein M1 is clearly present (Fig 2) in the non-split products and in the ether split formaldehyde inactivated product (5.1FE). The BPL inactivated ether split product (5.1BE) and the Triton split products (5.1FT and 5.1BT) contain only a light M1 band. In the preceding 3.1F and 3.1B fractions (before splitting) and after removal of Triton, however, M1 is still present (Fig 3). Apparently, M1 is removed during sterile filtration, when large complexes are retained on the filter. This observation is supported by DLS results (see next paragraph) of BPL inactivated product after splitting with Triton and removal of Triton, before (3.3BT) and after sterile filtration (5.1BT); less large entities are present in the product after sterile filtration (Fig 4, right panel, DLS results of fraction BT before and after sterile filtration)). The observation that M1 is present in only small amounts in split influenza vaccine, compared to the amount of M1 in whole virus vaccines, was also described by Chaloupka [26], when comparing vaccine products commercially available in Europe in the 1990’s.

### Characterization and comparison of (intermediate) product fractions by DLS and EM

The size of the different (intermediate) products was measured by DLS; results are presented in Table 6.

**Table 6:** DLS results of products before and after split and before and after sterile filtration (SF). The radius of the particles was measured before and after splitting (if applicable) and before and after SF. The results of the BPL inactivated fractions are given in the columns to the right, while the results of the formaldehyde inactivated fractions are presented in the columns to the left.

	Formaldehyde inactivated products				BPL inactivated products			
Fraction	Code (Fig 1)	r.nm	r, SD	PDI	Code (Fig 1)	r.nm	r, SD	PDI
Before split	3.0	78	28	0.13	3.1B	81	30	0.10
After split ether	3.3FE	77	34	0.22	3.3BE	64	39	0.22
After SF	5.1FE	64	28	0.18	5.1BE	52	22	0.18
Before split	3.1F	74	26	0.11	3.1B	81	30	0.10
After split Triton	3.3FT	83	47	0.19	3.3BT	81	42	0.27
After SF	5.1FT	54	28	0.28	5.1BT	66	32	0.23
Before SF	3.1F	74	26	0.11	3.1B	81	30	0.10
After SF	5.1F	82	25	0.11	5.1B	78	21	0.04

DLS analysis of whole virus (fractions 3.0, 3.1F and 3.1B) revealed a rather homogeneous size distribution of the virus (Table 6), as also demonstrated in Fig 4, left panel.

The mean size before and after splitting did not significantly change, however the PDI did. Especially the PDI of BPL inactivated product (3.1B) increased significantly upon splitting by Triton (3.3BT): from 0.10 to 0.27 (Table 6). The increase of size distribution and the two populations in the DLS graphs (Fig 4, right panel) indicate that the splitting of the virus with detergent did change its morphology.

By performing 0.22 μm sterile filtration (SF), large particles are removed from the intermediate influenza vaccine product as shown by the overlaid DLS graphs (Fig 4, right panel) of the BPL inactivated product before (3.3BT) and after SF (5.1BT). Due to the fact that large particles contribute more strongly to the signal, the removal of large particles seemingly results in a shift of the size to particles smaller than 200 nm. However, these small particles are also present in the material before filtration but are not detected. The shift may also be due to disintegration of large particles caused by shear force during sterile filtration.

**Fig 5:**
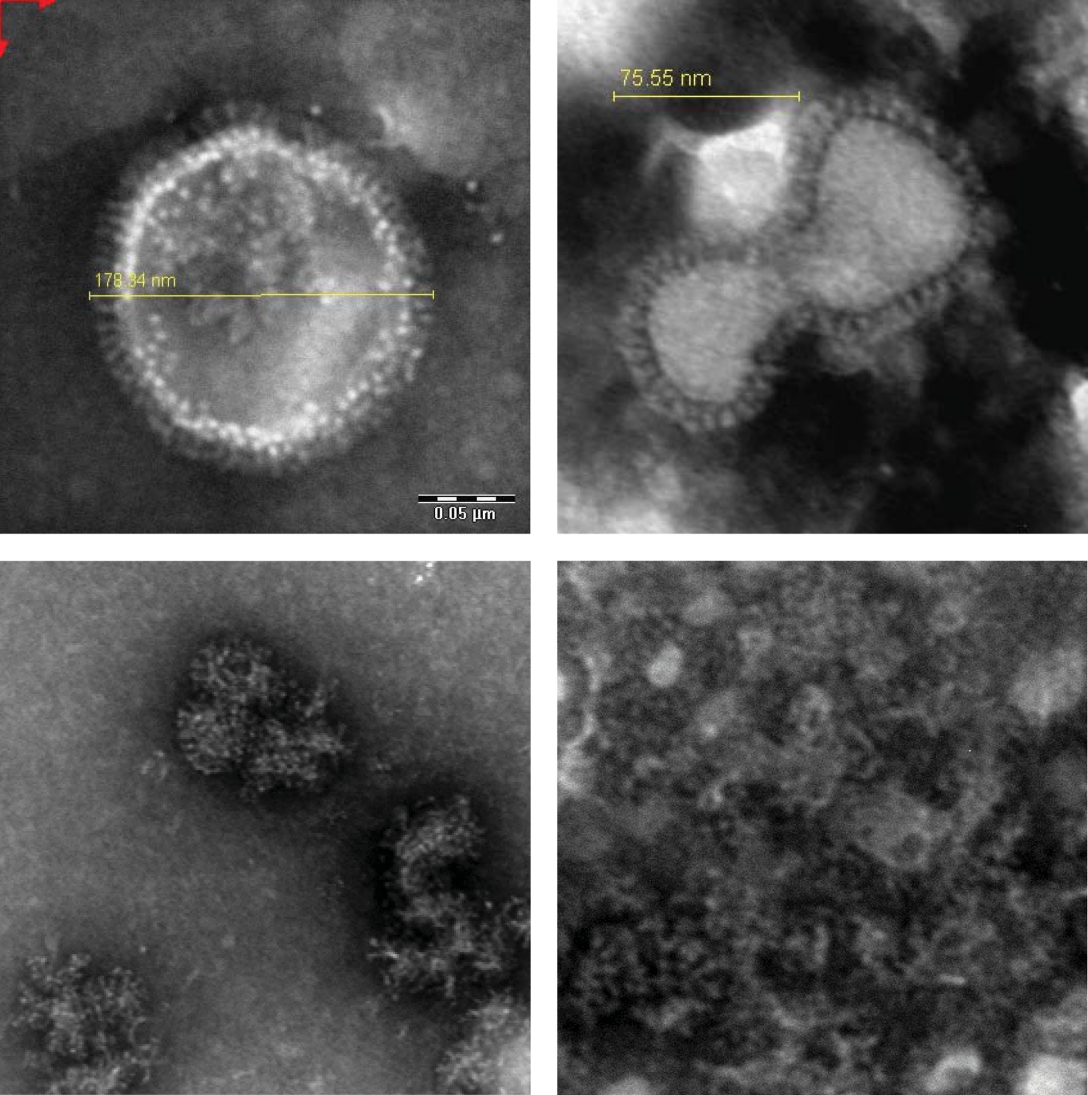
Representative electron microscope pictures of influenza virus particles, before and after split. Enlargement pictures to the left 300.000x, pictures to the right 400.000x.Top row whole virus (Fig 1, fraction 3.0), bottom row left panel ether split formaldehyde inactivated virus (Fig 1, fraction 5.1FE), bottom right panel BPL inactivated Triton split virus (Fig 1, fraction 5.1BT). Pictures at top: HA and NA spikes are clearly visible on the outside of the particles. The pictures at the bottom show disrupted, heterologous structures.

EM pictures of the H3N2 influenza virus, before splitting (Fig 5, top panels) show the presence of the spike proteins HA and NA and the particulate nature of the virus. The EM pictures of the inactivated and split H3N2 influenza (Fig 5, bottom panels) clearly show the partially disrupted particular and more heterologous structures, supporting the increase of PDI mentioned above.

### Stability of vaccine bulk products

**Fig 6:**
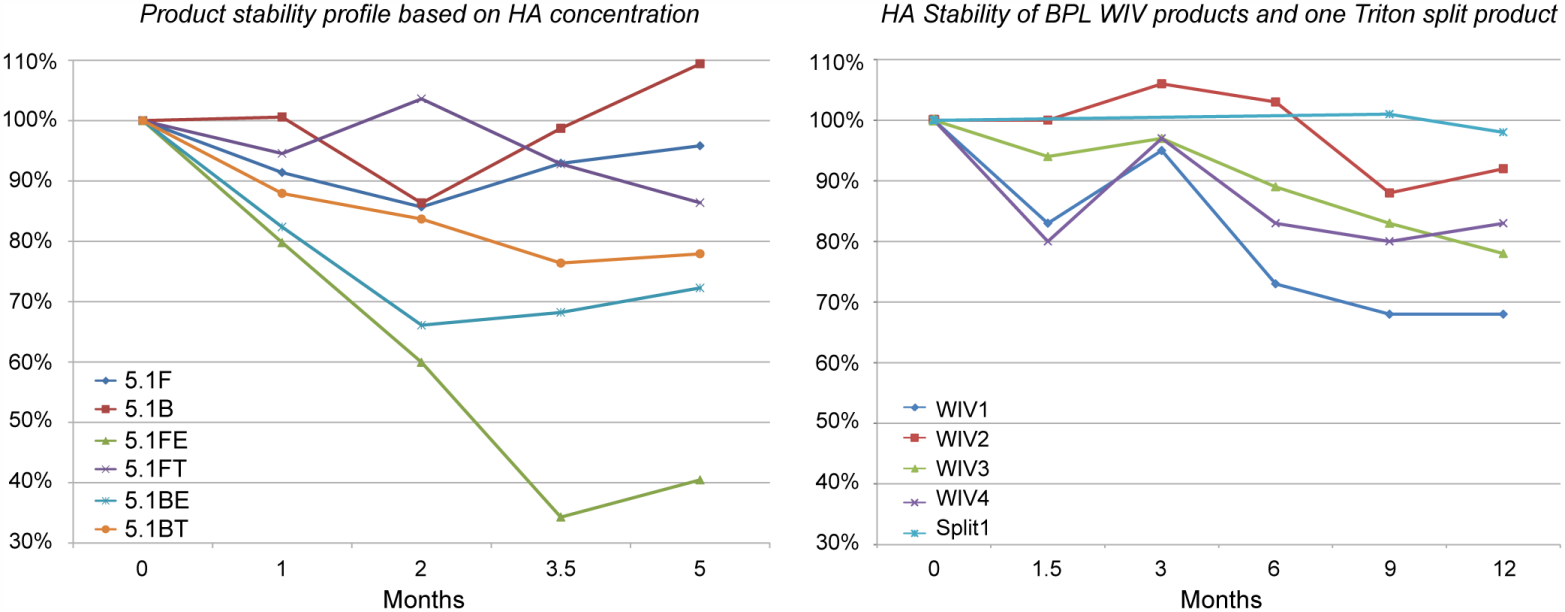
Graphs presenting the stability of vaccine bulk products, based on haemagglutinin concentration. Y-axis: HA μg/mL by SRID at t = 0 is 100%; X-axis: duration in months. The left graph presents the six vaccine bulks over a period of 5 months. Given the HA test variation of 20% and the limited data set, it can be concluded that the ether split formaldehyde inactivated product 5.1FE has least stability. The right graph presents data from influenza vaccine batches prepared at Intravacc (inactivation with BPL and splitting with Triton): stability over a period of twelve months for four WIV products and one Triton split product. The product stabilities are in the same range as in the left panel except for product 5.1FE.

Stability of the bulk products at 2–8°C was investigated over a period of five months by monitoring HA (SRID) content; the result is presented in Fig 6, left panel.

Based on the HA concentration, WIV products 5.1B and 5.1F seem to show slightly better stability than the average of the split products. Lowest stability was found for the ether split, formaldehyde inactivated product (5.1FE): it’s HA concentration decreased to half in 5 months. Given the limited data set and the HA test variation of 20% results are indicative only

For comparison the HA concentration data over a period of one year from four BPL inactivated H3N2 Uruguay WIV batches produced at Intravacc is presented in Fig 6, right panel. These batches are prepared similarly to product 5.1B manufactured at Cantacuzino. The stability data of an investigational batch, BPL inactivated Triton split product, (similar to at Cantacuzino produced 5.1BT, but with batch wise removal of Triton instead of recirculation over a packed column) showed high stability. The stability study of trivalent subunit influenza vaccine batches by Coenen e.a [27], including A/New Caledonia and/or A/Panama stored at 5°C+/- 3°C, showed a HA concentration trend line slope estimation of -0.030 and -0.094 respectively, indicating similar stability of this sub unit vaccines and BPL inactivated Triton split H3N2 bulk (5.1BT).

## General Discussion

In this study six downstream processes for influenza vaccine manufacturing were executed (Fig 1) and compared. Mostly equipment for industrial scale was used to facilitate later technical transfer of a revised DSP manufacturing process. Two WIV bulks and four split influenza vaccine bulks were manufactured. The bulks were analyzed using a panel of assays (Table 4), as defined by existing specifications for influenza vaccine product release. Additional testing of (intermediate) products was performed to support better control over product and downstream process.

For the release of influenza vaccine the prime focus is on the presence of antigenic HA, as determined with SRID, and the absence of specific contaminants, such as ovalbumin. The broad range in effective dose expressed in HA content from 3 to 45 μg per HA subtype in approved influenza vaccine products [28] seems to underline the effect of other product properties, such as the presence of NA and M1. For example Cox e.a [29] published that antibodies against M1 play a role in the clearance of virus and recovery from illness

The effect of detergents or organic solvents used for the disruption of influenza viruses depends on the quality of the input virus. Product properties such as the presence of detergent resistant membrane structures [30] and the presence of one ribonucleoprotein structure in either small spheroid or large filamentous virus [31] will affect the quality of the intermediate vaccine product. The used agent for inactivation can as well influence product quality [32] because formaldehyde performs cross-linking between molecular structures [33] and BPL causes acylation and alkylation of molecules [34].

SDS-PAGE revealed bands (Fig 2) at molecular weight of NP (55–60 kD) and NA (52 kD) in all products, except in the sterile filtered formaldehyde inactivated Triton split product 5.1FT (Fig 1). It is surprising to observe an apparent greater decease in M1, the protein that connects with all viral components and the membrane [30,35], while HA and NP are retained. No specific explanation for this can be given yet, though detergents will not affect the HA and NA associated in lipid rafts but do interfere with other binding sites given the observed effects of detergent on split vaccine product morphology [36]. M1 has a strong hydrophobic core and it may form dimers when in solution. In addition M1 dimers may stack up to a ribbon, all with their positively charged area on the same side of the ribbon [37]. If this causes larges entities, these are removed during the downstream process, especially during sterile filtration.

With respect to the presence of NP which was identified in pandemic vaccine formulations related to narcolepsy, as published recently by Vaarala [35], the manufacturing process using formaldehyde inactivation and Triton splitting may possibly result in a safer product.

### Acceptance criteria

All but one bulk, fulfill the preset criteria (Table 2), the only exception being the ether split, formaldehyde inactivated A/Uruguay/H3N2 product (5.1FE). The main reason is the finding that the HA concentration was too low. The HA concentration as measured by SRID in 5.1FE product is lower than the mean value found at Cantacuzino during routine manufacturing using the same strain and the same DSP process. The variation in the SRID assay was determined at 20%, which implies that the single value for this bulk could still be within specification. Results from SDS-PAGE of de-glycosylated samples and densitometry using samples from this bulk show that the HA1-protein band is in the same range of intensity as the HA1 bands of the other bulk products, indicating that part of HA is not recognized by antibodies in the SRID test.

### Recovery and stability

HA recoveries after the main unit operations reveal that in all processes substantial (15–50%) losses occurred during sterile filtration. This can be explained by the fact that the virus and the split virus particles are relative large entities compared to the pore size of the membrane used for SF. After SF still particles are present with size of 200 nm (radius of 100 nm) and larger, possibly indicating an ongoing association process or equilibrium.

Smaller HA losses are observed after splitting and removal of the chemical agent in the case of ether splitting of the BPL inactivated product and the Triton splitting of the formaldehyde inactivated product: a decrease of circa 15% HA. In the other processes HA recovery after splitting and removal of the chemical agent was 96% (ether split) and 91% (BPL inactivated, Triton split). Using these unit operations the protein content decreased even more than the antigenic HA content (except for process of ether split, formaldehyde inactivation, where the order of the unit operations is different). Based on these limited HA recovery data, on average processes with BPL inactivation gave higher recoveries.

Of the six different preparations, the ether split formaldehyde inactivated A/Uruguay/H3N2 bulk product (5.1FE) showed least stability. Whole inactivated A/Uruguay/H3N2 virus bulks appear to have good stability, possibly the structure of the whole virus protects its integrity.

Commonly it is hypothesized that the presence of a certain amount of detergent improves the product stability; Triton X-100 is intentionally added [38] and present in most of the commercial products. The majority of the products investigated here are exceptionally stable, despite the (almost) complete detergent removal.

### Comparison of batches produced at different sites

Recovery and composition of the bulk product produced at Cantacuzino Institute Romania during process transfer, with the inactivation using BPL and splitting using Triton, is compared with the average of 6 batches produced in The Netherlands at Intravacc for clinical studies. In Table 7 the main result of the comparison is presented. SDS-PAGE results of bulks presented in Fig 2 confirm the principal protein composition resemblance of products produced at either location. Therefore as proof-of-concept the process transfer can be considered successful for this one batch.

**Table 7:** Overview of the main characteristics of the BPL inactivated, Triton split bulk produced at Cantacuzino Institute, Romania and the average of clinical batches produced at Intravacc, The Netherlands.

		Romania Cantacuzino	The Netherlands Intravacc	
Parameter, Quality attribute	Requirement [unit]	n = 1	Average, n = 6	StDev, n = 6
**HA**	> 90 [μg/mL]	333	160	62
**HA/total protein**	[μg/100 μg TP]	44	44	4.8
**total protein/HA**	≤ 600 [μg/100 μg HA]	227	229	23
**ovalbumin/HA**	≤ 2 [μg/100 μg HA]	0.9	0.003	0.003
**Recovery HA**	[%]	64%	53%	13%

**Notes**: HA concentration based on SRID test. The product prepared in Romania is more concentrated (products produced in The Netherlands were diluted 1:1 during SF, while Romania product was not), contained more residual ovalbumin and had higher HA recovery. The product prepared at Intravacc has a lower residual ovalbumin content; eggs are selected for similar size and the allantoic fluid is harvested in a very precise mode, preventing contamination with e.g. albumen, egg yolk, amniotic fluid and serum of fertilized eggs.

## Conclusions

All influenza vaccine bulk products produced at Cantacuzino met the preset quality criteria (WHO, EP), with respect to sterility, presence of NA and maximum ovalbumin content. However, the HA content of the ether-split formaldehyde-inactivated product as determined by SRID was found to be lower than the specification; this single value was still within the standard variation of the SRID assay of 20%. This study shows the initial feasibility of process transfer of different influenza DSP processes, as a potential first step towards implementation of a revised manufacturing process, using additional product characterization to support better control over product and process.

SDS-PAGE revealed that NP was (nearly) absent in the product inactivated by formaldehyde and split by Triton and M1 largely disappeared from the split products.

Monitoring the HA content of bulk vaccine stored at 2–8°C for 5 months, the ether-split formaldehyde-inactivated product showed a major loss of more than 50%, while the Triton split formaldehyde-inactivated product had a HA a loss of only 13%.

Recovery of the processes, based on HA measured by SRID, ranged from 27% to 81%. Largest losses occurred during the sterile filtration unit operation. The recovery over sterile filtration step only, ranged from 32% (ether split formaldehyde inactivated product) to 90% (BPL inactivated, ether split product). EM and DLS results show the removal of larger structures, which is correlated with the reduction of M1 content in intermediate product as shown with SDS-PAGE.

WIV products also suffered from substantial losses after sterile filtration: HA recovery of 49% (formaldehyde inactivated product) and 71% (BPL inactivated product). The shape of the virus (longer than wide) may contribute to (partial) blockage of the sterile filter membrane.

On average BPL inactivated virus product show higher recovery than the formaldehyde treated products. The overall recoveries of the Triton split products, whether BPL inactivated or formaldehyde inactivated, seem to be similar. WIV products and formaldehyde inactivated Triton split product show better stability in this study based on limited data.

This investigation confirmed the influence of choices made for the downstream process on the final product quality, recovery and stability.

## Supporting Information

S1 Fig Coomassie Brilliant Blue stained SDS-PAGE gels with different fractions (Fig 1) of the influenza vaccine downstream processes and marker proteins. Lanes numbered above left gel: 1.1 allantoic fluid, 1.2 clarified allantoic fluid, 3.0 purified virus, 3.1F formaldehyde inactivated virus, 5.1F sterile filtered bulk product of formaldehyde inactivated virus, 5.1FE sterile filtered bulk product of formaldehyde inactivated ether split virus, 5.1FT sterile filtered bulk product of formaldehyde inactivated Triton split virus, M marker proteins.
Lanes numbered above right gel: 1.1 allantoic fluid, 1.2 clarified allantoic fluid, 3.0 purified virus, 3.1B beta-propiolactone (BPL) inactivated virus, 5.1B sterile filtered bulk product of BPL inactivated virus, 5.1BT sterile filtered bulk product of BPL inactivated Triton split virus, 5.1BE sterile filtered bulk product of BPL inactivated ether split virus, M marker proteins.
Fractions 3.0 (whole virus), 3.1F (formaldehyde inactivated whole virus) and 3.1B (BPL inactivated whole virus) evaluated by SDS-PAGE resemble the WIV product fractions 5.1F and 5.1B; no major change in protein composition due to inactivation or sterile filtration is visible.
(TIF)

S1 File Analyses methods used for testing the main characteristics of the influenza fractions (PDF)

S2 File Detection of unexpected discrepancies between the proportional decrease in protein and the proportional decrease in the quantity of virus following a given step in the production process. Statistical analysis of recovery results.
(PDF)
