# Supplementary figures and images for: Influenza Vaccine Manufacturing: Effect of Inactivation, Splitting and Site of Manufacturing. Comparison of Influenza Vaccine Production Processes
Source: PLoS One. 2016 Mar 9;11(3):e0150700. doi: 10.1371/journal.pone.0150700 (PMC4784929; doi:10.1371/journal.pone.0150700)

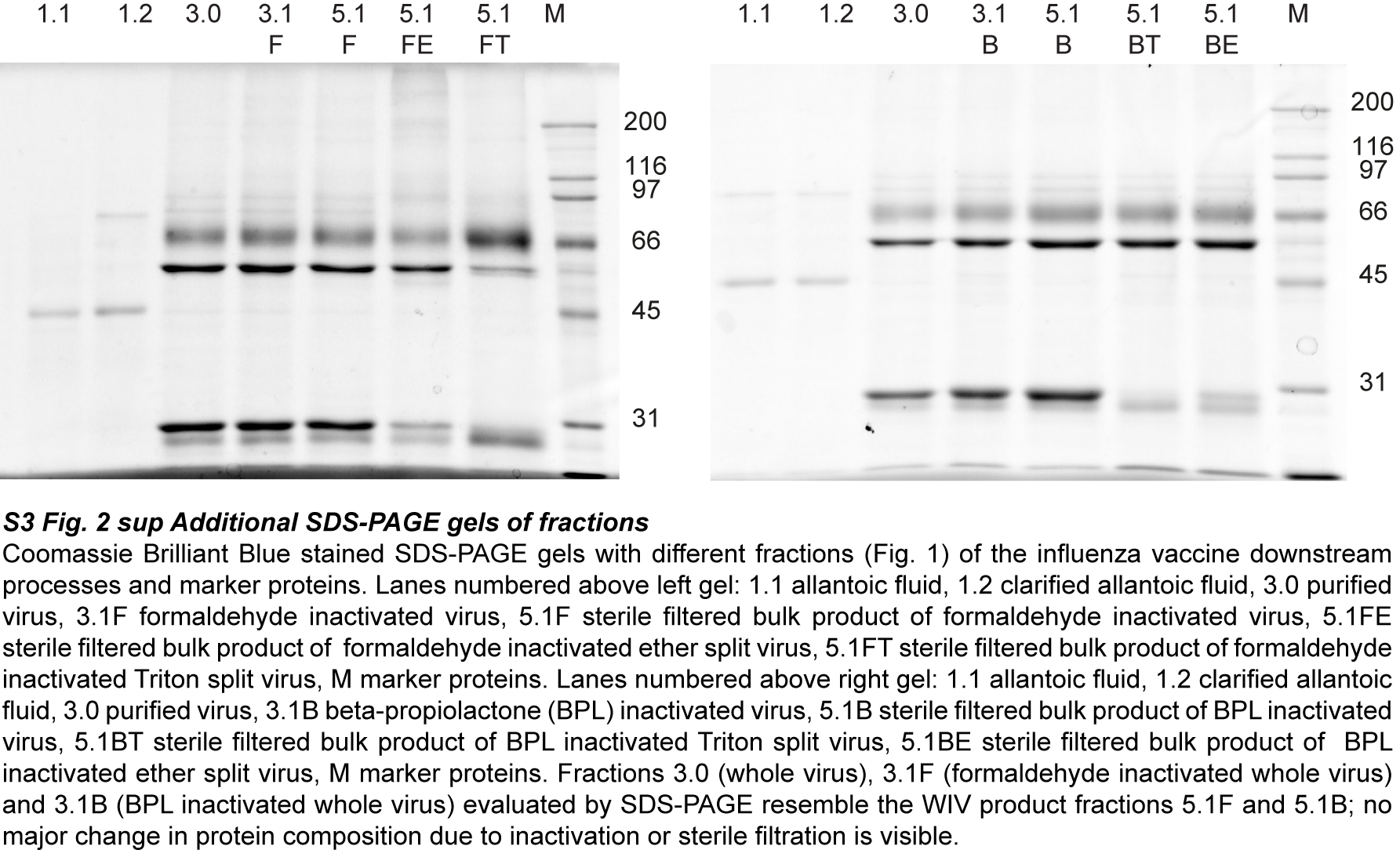

Supplement: S1 Fig — Lanes numbered above left gel: 1.1 allantoic fluid, 1.2 clarified allantoic fluid, 3.0 purified virus, 3.1F formaldehyde inactivated virus, 5.1F sterile filtered bulk product of formaldehyde inactivated virus, 5.1FE sterile filtered bulk product of formaldehyde inactivated ether split virus, 5.1FT sterile filtered bulk product of formaldehyde inactivated Triton split virus, M marker proteins. Lanes numbered above right gel: 1.1 allantoic fluid, 1.2 clarified allantoic fluid, 3.0 purified virus, 3.1B beta-propiolactone (BPL) inactivated virus, 5.1B sterile filtered bulk product of BPL inactivated virus, 5.1BT sterile filtered bulk product of BPL inactivated Triton split virus, 5.1BE sterile filtered bulk product of BPL inactivated ether split virus, M marker proteins. Fractions 3.0 (whole virus), 3.1F (formaldehyde inactivated whole virus) and 3.1B (BPL inactivated whole virus) evaluated by SDS-PAGE resemble the WIV product fractions 5.1F and 5.1B; no major change in protein composition due to inactivation or sterile filtration is visible. (TIF) [file pone.0150700.s001.tif]
