# Supplementary material for: Influenza Vaccine Manufacturing: Effect of Inactivation, Splitting and Site of Manufacturing. Comparison of Influenza Vaccine Production Processes
Source: PLoS One. 2016 Mar 9;11(3):e0150700. doi: 10.1371/journal.pone.0150700 (PMC4784929; doi:10.1371/journal.pone.0150700)
Supplement: S1 File — (PDF) [file pone.0150700.s002.pdf]

S1 Methods

Analyses methods used for testing the main characteristics of the influenza fractions

SRID

Haemagglutinin quantification and virus identity was performed using SRID (Single Radial Immuno Diffusion) assay.

SRID assay is based on the reaction between the antibodies present in a flat agarose gel and the antigen that diffuses from an application spot in the gel. Once the concentration of antibody and antigen are equal a precipitation in the shape of a ring occurs. The precipitate was stained using Coomassie Brilliant Blue and the size of the ring was used as a measure for the concentration [1].

Materials:

Agarose Indubiose A37, Biosepra Art. 23023-021, Gelbond-film Lonza.

Influenza anti A/Brisbane/10/2007- like HA serum NIBCS code 08/246.

Influenza Antigen A/Brisbane/10/2007-like (Prepared from A/Uruguay/716/2007 (NYMC X-175C)) NIBSC code 08/278.

[1] J. M. Wood, G. C. Schild, R. W. Newman, and Valerie Seagroatt, An improved single-radialimmunodiffusion technique for the assay of influenza haemagglutinin antigen: application for potency determinations of inactivated whole virus and subunit vaccines, Journal of Biological Standardization 1977 5, 237-247

Total protein

Total protein quantity was determined by using the Peterson colorimetric method [2]. (Membrane bound) proteins were first dissolved with deoxycholate, then the proteins were precipitated using trichloric acid and subsequently Lowry assay was performed.

Materials

Folin-Ciocaleu's Phenol Reagent 2 N, Sigma F-9252, Spectrophotometer

[2] Peterson G.L., A simplification of the protein assay of Lowry et al, which is more generally applicable. Anal. Biochem. 1977, 83, p.346-356.

Ovalbumin

Ovalbumin concentration was measured using a direct sandwich ELISA (Enzyme Linked Immuno Sorbent Assay).

The assay was performed according to the instructions of the supplier of the ELISA kit. Independent duplicates of two different dilutions were uses as samples. Samples containing the antigen were pipetted in ELISA plate wells coated with polyclonal anti-ovalbumin antibodies. Anti-ovalbumine-linked to Horse Radish Peroxidase was added, followed by washing away unbound substances. The addition of a substrate initiated the development of a blue color. This process was stopped by adding sulphoric acid; the color changed from blue to yellow. The absorption at 450nm was a measure for the quantity. A 630nm filter was used as reference.

Materials

Serazym Ovalbumin ELISA: Cat.No. E041C, Seramun Diagnostica GmbH Wolzig.

ELISA-plate reader with filter 450nm and reference filter 630nm

Endotoxin

Endotoxin content was measured using the de Limulus Amoebocyte Lysate (LAL) test. Small amounts of endotoxine cause clotting of a of amoebocyte lysate from horseshoe crab. The test is semi quantitative and described in the European Pharmacopoeia [3].

The lysate sensitivity (λ) mentioned in the analysis certificate was confirmed with standard solutions. Equal amounts of lysate were added to multiple dilutions of the sample. To verify that the sample matrix is of no influence, samples were also spiked with the standard and tested. The verification samples should clot. After incubation at 37°C bromothymol blue was added to facilitate reading: if no clotting the stain was distributed equally and in case of a gel clot the stain solution flows to one side of the well.

Materials
Limulus Amoebocyte Lysate (LAL), CSE Standard, LAL Reagent Water (LRW), pipettes, tubes, microtiter plates all from Charles River Endosave
[3] European Pharmacopoeia 2.6.14, Bacterial Endotoxins Gel-clot Technique (method B). European Pharmacopoeia 5.1.10, Guidelines for using the test for bacterial endotoxins.

Neuraminidase inhibition assay
The purpose of this test is to determine the presence and identity of neuraminidase in master seed virus (MSV), working seed virus (WSV) and monovalent bulk vaccine (MBV) using the neuraminidase activity and inhibition test.
The test is based on the following principle: neuraminidase can release sialic acid from the substrate fetuïn. This sialic acid can react to build a colored complex, which is measured spectrophotometrically. The concentration of the antigen which gives an Optical Density (OD) of approximately 0.8 is used for the NI-test. In this test antisera, specific to A- (N_1 and N_2) and B- (NB) strains, are used to give an interaction with the present antigen, thereby inhibiting the development of a colored complex. Present antigens are bound to specific antibodies. Remaining unbound neuraminidase results, after releasing sialic acid, in a colored complex. The NI titer is defined as the serum dilution whereby 50% of neuraminidase activity is inhibited.
Materials
Fetuin, Neuraminidase Antiserum N_1, Neuraminidase Antiserum N_2, Neuraminidase Antiserum B, Control Sheep Antiserum (CS).

Residual Infectious Viruses
The purpose of this test is to confirm the absence of any residual infectious influenza viruses in the monovalent bulk vaccine (MBV) after treatment with beta-propiolactone (BPL).
For each MBV, 10 fertilized eggs will be inoculated with 0.2 ml MBV into the allantoic cavity. After 3 days incubation, 0.5 ml allantoic fluid is harvested from the eggs and pooled. Again 10 fertilized eggs will be inoculated with 0.2 ml allantoic fluid from the 1st pooled harvest. After 3 days incubation, the allantoic fluids are harvested and incubated with a fixed amount of turkey erythrocytes. If there are viruses present in the allantoic fluids, haemagglutination with the erythrocytes will take place. If haemagglutination is found, further passage in eggs will be carried out.
Materials
11 days old fertilized chicken eggs, Turkey blood, Bovine Serum Albumin

Sterility
The purpose of this test is to determine the sterility of master seed lot (MSL), working seed lot (WSL) and monovalent bulk vaccine (MBV).
The sample is filtrated through a membrane, flushed and placed in media. After incubation the media is examined for turbidity, which indicates the presence of micro-organisms. The advantage of filtration of the sample is based on removing factors that could inhibit the growth of micro-organisms. Furthermore, a bigger volume of sample can be tested for the presence of micro-organisms which increases the sensitivity of the test.
Materials
Pseudomonas aeruginosa
Baccilus subtilis
