# Supplementary material for: Influenza Vaccine Manufacturing: Effect of Inactivation, Splitting and Site of Manufacturing. Comparison of Influenza Vaccine Production Processes
Source: PLoS One. 2016 Mar 9;11(3):e0150700. doi: 10.1371/journal.pone.0150700 (PMC4784929; doi:10.1371/journal.pone.0150700)

S2 **Detection of unexpected discrepancies between the proportional decrease in protein and the proportional decrease in the quantity of virus following a given step in the production process**
Statistical analysis of recovery results

Our data consist of proportional decreases in the quantity of protein and in the quantity of haemagglutinine (HA) in the product after each step of each of six production processes:

	process	step	fraction	protein	HA	log.ratio
1	ether split virus, FE	after zonal	2.1	1.00	1.00	0.00000000
2	ether split virus, FE	after inactivation	2.2	NA	NA	NA
3	ether split virus, FE	after DF	3.0,3.1	0.87	0.86	0.01156082
4	ether split virus, FE	after split	3.3	0.94	0.96	-0.02105341
5	ether split virus, FE	after SF	5.1	0.72	0.32	0.81093022
6	whole virus, F	after zonal	2.1	1.00	1.00	0.00000000
7	whole virus, F	after inactivation	2.2	1.15	0.95	0.19105524
8	whole virus, F	after DF	3.0,3.1	0.93	1.04	-0.11179141
9	whole virus, F	after split	3.3	NA	NA	NA
10	whole virus, F	after SF	5.1	0.52	0.49	0.05942342
11	Triton split virus, FT	after zonal	2.1	1.00	1.00	0.00000000
12	Triton split virus, FT	after inactivation	2.2	1.15	0.95	0.19105524
13	Triton split virus, FT	after DF	3.0,3.1	0.93	1.04	-0.11179141
14	Triton split virus, FT	after split	3.3	0.70	0.85	-0.19415601
15	Triton split virus, FT	after SF	5.1	0.49	0.72	-0.38484582
16	ether split virus, BE	after zonal	2.1	1.00	1.00	0.00000000
17	ether split virus, BE	after inactivation	2.2	1.03	1.03	0.00000000
18	ether split virus, BE	after DF	3.0,3.1	1.00	1.02	-0.01980263
19	ether split virus, BE	after split	3.3	0.68	0.85	-0.22314355
20	ether split virus, BE	after SF	5.1	0.84	0.90	-0.06899287
21	whole virus, B	after zonal	2.1	1.00	1.00	0.00000000
22	whole virus, B	after inactivation	2.2	1.03	1.03	0.00000000
23	whole virus, B	after DF	3.0,3.1	1.00	1.02	-0.01980263
24	whole virus, B	after split	3.3	NA	NA	NA
25	whole virus, B	after SF	5.1	0.69	0.71	-0.02857337
26	Triton split virus, BT	after zonal	2.1	1.00	1.00	0.00000000
27	Triton split virus, BT	after inactivation	2.2	1.03	1.03	0.00000000
28	Triton split virus, BT	after DF	3.0,3.1	1.00	1.02	-0.01980263
29	Triton split virus, BT	after split	3.3	0.77	0.91	-0.16705408
30	Triton split virus, BT	after SF	5.1	0.52	0.67	-0.25344890

A couple of steps in a couple of processes have yielded no data, indicated above by NA. The last column contains the natural logarithms of the ratio of the proportional decrease in protein to the proportional decrease in HA, referred to in the sequel as *log-ratios*. If we remove the missing data and the initial values of 100% of each product we get a data set with 21 observations:

	process	step	fraction	protein	HA	log.ratio
1	ether split virus, FE	after DF	3.0,3.1	0.87	0.86	0.01156082
2	ether split virus, FE	after split	3.3	0.94	0.96	-0.02105341
3	ether split virus, FE	after SF	5.1	0.72	0.32	0.81093022
4	whole virus, F	after inactivation	2.2	1.15	0.95	0.19105524
5	whole virus, F	after DF	3.0,3.1	0.93	1.04	-0.11179141
6	whole virus, F	after SF	5.1	0.52	0.49	0.05942342
7	Triton split virus, FT	after inactivation	2.2	1.15	0.95	0.19105524
8	Triton split virus, FT	after DF	3.0,3.1	0.93	1.04	-0.11179141
9	Triton split virus, FT	after split	3.3	0.70	0.85	-0.19415601
10	Triton split virus, FT	after SF	5.1	0.49	0.72	-0.38484582
11	ether split virus, BE	after inactivation	2.2	1.03	1.03	0.00000000
12	ether split virus, BE	after DF	3.0,3.1	1.00	1.02	-0.01980263
13	ether split virus, BE	after split	3.3	0.68	0.85	-0.22314355
14	ether split virus, BE	after SF	5.1	0.84	0.90	-0.06899287
15	whole virus, B	after inactivation	2.2	1.03	1.03	0.00000000
16	whole virus, B	after DF	3.0,3.1	1.00	1.02	-0.01980263
17	whole virus, B	after SF	5.1	0.69	0.71	-0.02857337

```
18 Triton split virus, BT after inactivation     2.2              1.03 1.03  0.00000000
19 Triton split virus, BT             after DF     3.0,3.1          1.00 1.02 -0.01980263
20 Triton split virus, BT          after split     3.3              0.77 0.91 -0.16705408
21 Triton split virus, BT             after SF     5.1              0.52 0.67 -0.25344890
```

In theory, if everything goes well during a given step of one of the six prediction processes then the proportional decrease in protein at the end of that step should be equal to the proportional decrease in virus content (denoted here by HA), so that, allowing for random (measurement) error, the corresponding log-ratio *should not be too far from zero*. We would like to detect 'irregular' situations where (for some reason) this expectation is not fulfilled.

This task is not straightforward because there is a single log-ratio for each combination of production step and process, and therefore it is impossible to estimate the variance of the log-ratios per combination of step and process. Moreover, we have no reason to believe that the variance is the same for every step and process. For example, the box-plots of the log-ratios per process indicate that the process "whole virus, B" may have smaller variances than the other five:

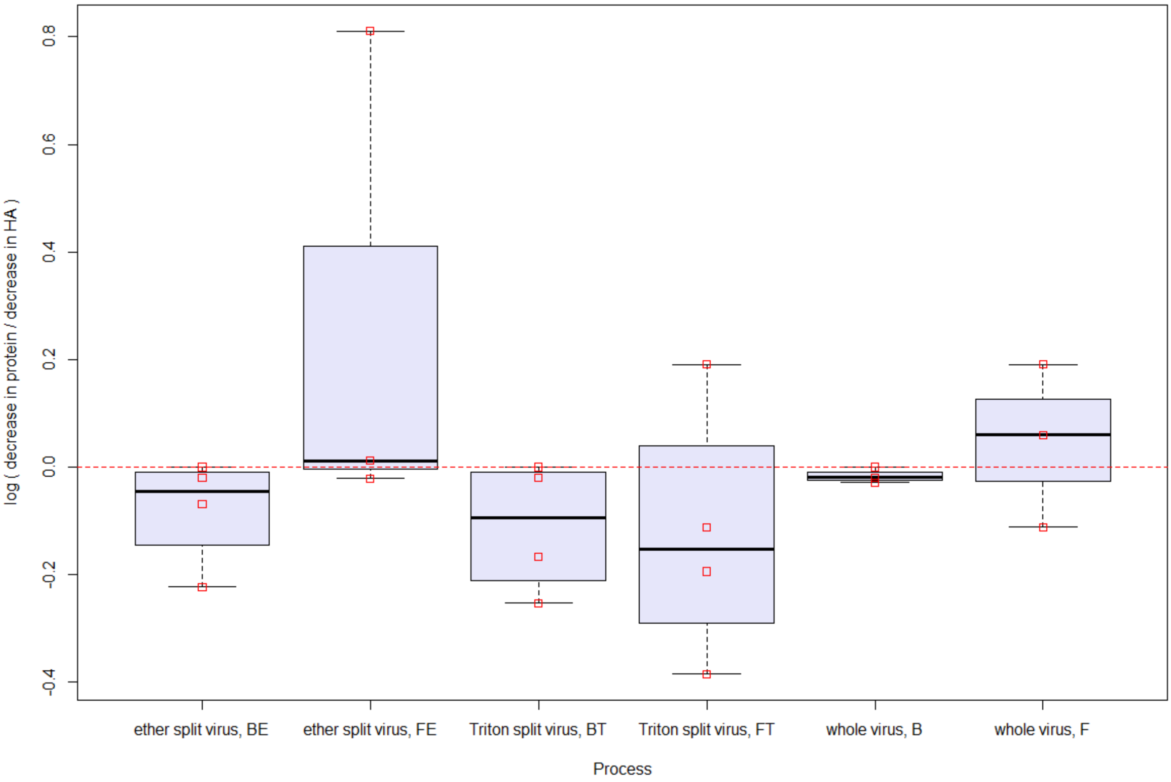

A conservative solution to our *problem of detection*, which nevertheless requires some assumptions, is as follows.

Assume that each log-ratio, denoted by $\log R_{s,p}$, measured following step s of a process p is normally distributed with mean 0 and standard deviation $\sigma_{s,p}$. The assumption of a zero mean reflects the expectation that the decreases in the amount of protein and in the amount of virus are equal. The normality assumption is plausible because random proportional decreases tend to be log-normally distributed, but it cannot be checked on the basis of our data. If $\sigma_{s,p}$ were known, a p-value could be computed as

$$P_{s,p} := \Phi\left(-\frac{|\log R_{s,p}|}{\sigma_{s,p}}\right) + 1 - \Phi\left(\frac{|\log R_{s,p}|}{\sigma_{s,p}}\right),$$

where as usual Φ denotes the standard normal distribution function, and used as evidence for an observed log-ratio $\log R_{s,p}$ being too far away from zero. Because $\sigma_{s,p}$ is unknown, we need to substitute $P_{s,p}$ by

$$P'_{s,p} := \Phi\left(-\frac{|\log R_{s,p}|}{\sigma}\right) + 1 - \Phi\left(\frac{|\log R_{s,p}|}{\sigma}\right),$$

for example, where σ *overestimates* $\sigma_{s,p}$. The rationale for doing this is that if $P'_{s,p}$ is 'small' then so is $P_{s,p}$, so if we find evidence based on $P'_{s,p}$ against the null hypothesis (that the decrease is identical in the protein content and in the virus content) then the corresponding evidence based on $P_{s,p}$ is at least as strong.—Our solution is conservative because the reverse implication need not hold: even if the unobservable evidence based on $P_{s,p}$ is strong, the 'conservative p-value' $P'_{s,p}$ will not necessarily warn us of that; consequently, our method may spot some of the bigger discrepancies regarding the expectations (that the decrease is identical in the protein and in the virus), but it may miss a few less conspicuous ones.

In order to find an appropriate value for σ, we compute robust estimates of a standard deviation, namely the *mad* (median absolute deviation), from the six samples of log-ratios corresponding to the six production processes:

	process	estimate.of.SD
1	ether split virus, BE	0.05114442
2	ether split virus, FE	0.04835386
3	Triton split virus, BT	0.12383719
4	Triton split virus, FT	0.20241524
5	whole virus, B	0.01300351
6	whole virus, F	0.19515733

Clearly, there is quite some variation in the estimates, but if we take σ as their maximum then we should comfortably fall on the safe (conservative) side. Taking $\sigma = 0.20$ and performing the tests we get the following p-values and bounds on the FDR (false discovery rate):

	process	step	protein	HA	log.ratio	p.value	bound.FDR
1	ether split virus, FE	after SF	0.72	0.32	0.81093022	6.168498e-05	0.001295385
2	Triton split virus, FT	after SF	0.49	0.72	-0.38484582	5.726678e-02	0.601301239
3	Triton split virus, BT	after SF	0.52	0.67	-0.25344890	2.105248e-01	1.473673748
4	ether split virus, BE	after split	0.68	0.85	-0.22314355	2.702857e-01	1.418999816
5	Triton split virus, FT	after split	0.70	0.85	-0.19415601	3.374597e-01	1.417330726
6	whole virus, F	after inactivation	1.15	0.95	0.19105524	3.452321e-01	1.208312453
7	Triton split virus, FT	after inactivation	1.15	0.95	0.19105524	3.452321e-01	1.035696388
8	Triton split virus, BT	after split	0.77	0.91	-0.16705408	4.091991e-01	1.074147584
9	whole virus, F	after DF	0.93	1.04	-0.11179141	5.807514e-01	1.355086582
10	Triton split virus, FT	after DF	0.93	1.04	-0.11179141	5.807514e-01	1.219577924
11	ether split virus, BE	after SF	0.84	0.90	-0.06899287	7.332179e-01	1.399779541
12	whole virus, F	after SF	0.52	0.49	0.05942342	7.690851e-01	1.345898875
13	whole virus, B	after SF	0.69	0.71	-0.02857337	8.877418e-01	1.434044507
14	ether split virus, FE	after split	0.94	0.96	-0.02105341	9.171606e-01	1.375740941
15	ether split virus, BE	after DF	1.00	1.02	-0.01980263	9.220659e-01	1.290892307
16	whole virus, B	after DF	1.00	1.02	-0.01980263	9.220659e-01	1.210211537
17	Triton split virus, BT	after DF	1.00	1.02	-0.01980263	9.220659e-01	1.139022623
18	ether split virus, FE	after DF	0.87	0.86	0.01156082	9.544541e-01	1.113529754
19	ether split virus, BE	after inactivation	1.03	1.03	0.00000000	1.000000e+00	1.105263158
20	whole virus, B	after inactivation	1.03	1.03	0.00000000	1.000000e+00	1.050000000
21	Triton split virus, BT	after inactivation	1.03	1.03	0.00000000	1.000000e+00	1.000000000

These results indicate that the discrepancy found at step "after SF" of the process "ether split virus, FE is too large to be attributed to chance (measurement error). For the other, smaller discrepancies we cannot adduce evidence, though as explained that may be due to lack of power (a by-product of our conservative approach). [Note that the second p-value is close to 0.05, but our multiple testing procedure based on controlling the FDR—the Benjamini-Hochberg method—ensures that the corresponding discrepancy is *not* called significant.] The following plot of the log-ratios as functions of 'fraction' illustrate the discrepancy found in "ether split virus, FE", "after SF". Intuitively, the range of the variability of the log-ratios measured on the other processes does

not seem to explain the discrepancy found. Perhaps interesting is the observation that most of the log-ratios are *below* 0, when in theory they should be more symmetrically distributed around it; however, with these data we cannot provide any evidence that this observation is significant—if that were the case our approach to detecting discrepancies would be invalidated!

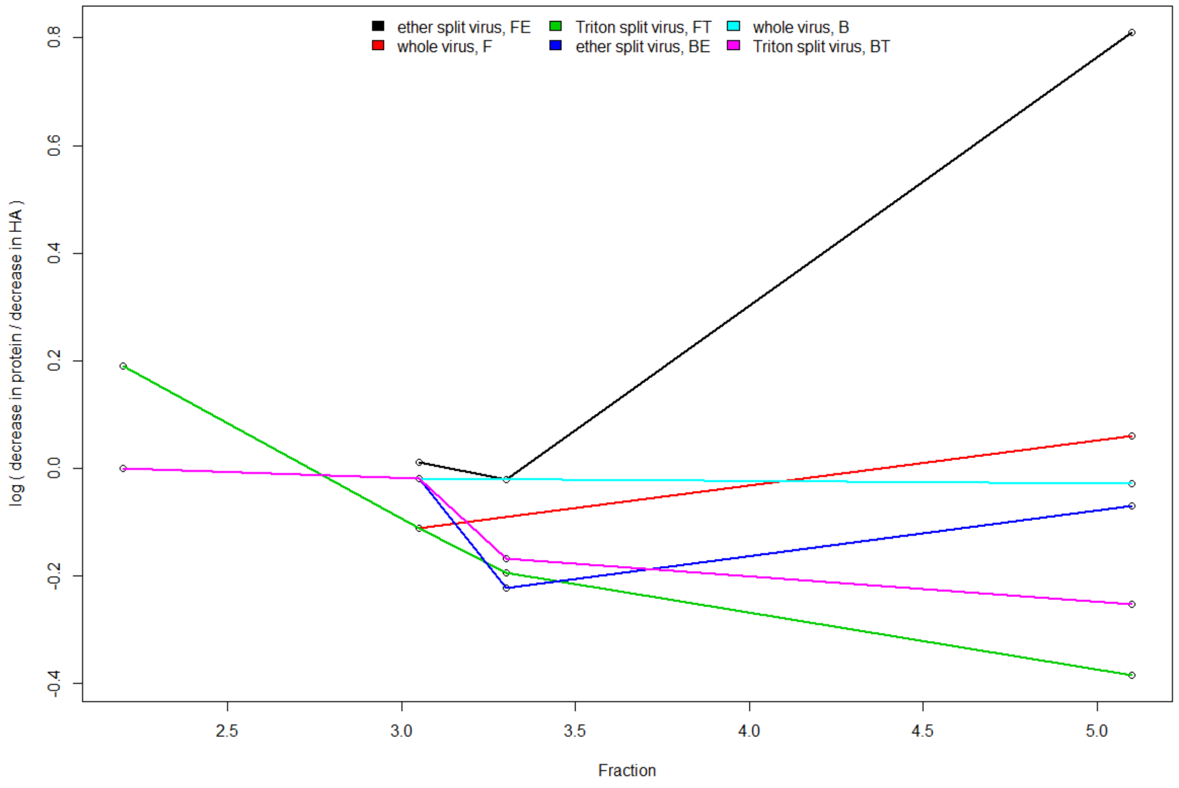

Supplement: S2 File — Statistical analysis of recovery results. (PDF) [file pone.0150700.s003.pdf]
